# Decomposition of peatland DOC affected by root exudates is driven by specific r and K strategic bacterial taxa

**DOI:** 10.1038/s41598-021-97698-2

**Published:** 2021-09-21

**Authors:** Jiří Mastný, Jiří Bárta, Eva Kaštovská, Tomáš Picek

**Affiliations:** Department of Ecosystem Biology, Faculty of Science, University of South Bohemia, Branišovská 1760, 37005 Ceske Budejovice, Czech Republic

**Keywords:** Bacteria, Microbial communities

## Abstract

In peatlands, decomposition of organic matter is limited by harsh environmental conditions and low decomposability of the plant material. Shifting vegetation composition from *Sphagnum* towards vascular plants is expected in response to climate change, which will lead to increased root exudate flux to the soil and stimulation of microbial growth and activity. We aimed to evaluate the effect of root exudates on the decomposition of recalcitrant dissolved organic carbon (DOC) and to identify microorganisms involved in this process. The exudation was mimicked by an addition of a mixture of ^13^C labelled compounds into the recalcitrant DOC in two realistic levels; 2% and 5% of total DOC and peatland porewater with added root exudates was incubated under controlled conditions in the lab. The early stage of incubation was characterized by a relative increase of r-strategic bacteria mainly from *Gammaproteobacteria* and *Bacteriodete*s phyla within the microbial community and their preferential use of the added compounds. At the later stage, *Alphaproteobacteria* and *Acidobacteria* members were the dominating phyla, which metabolized both the transformed ^13^C compounds and the recalcitrant DOC. Only higher exudate input (5% of total DOC) stimulated decomposition of recalcitrant DOC compared to non-amended control. The most important taxa with a potential to decompose complex DOC compounds were identified as: *Mucilaginibacter* (*Bacteriodete*s), *Burkholderia* and *Pseudomonas* (*Gammaproteobacteria*) among r-strategists and *Bryocella* and *Candidatus Solibacter* (*Acidobacteria*) among K-strategists. We conclude that increased root exudate inputs and their increasing C/N ratio stimulate growth and degradation potential of both r-strategic and K-strategic bacteria, which make the system more dynamic and may accelerate decomposition of peatland recalcitrant DOC.

## Introduction

In the peatlands, decomposition of soil organic carbon (SOC) is restricted due to prevailing anoxic conditions, low pH, nutrient limitation and low decomposability of the plant material. Consequently, peatland dissolved organic carbon (DOC) is also hardly decomposable and referred as recalcitrant^1^. Peatlands thus represent a substantial source of DOC to aquatic ecosystems^2^. Vascular plants and mosses release variety of organic compounds from their roots and steles including low molecular weight and easily decomposable exudates, such as organic acids, sugars and amino acids^3^. Such compounds stimulate microbial growth and activity^4^, including a production of extracellular enzymes, which may accelerate decomposition of peat and peatland DOC—a phenomenon called positive priming effect^5^. Positive priming effect has been reported in peatland ecosystems^6,7^, but its significance is considered as minor^7^ or unclear^6^. However, ongoing climate change is causing a spreading of vascular plants, namely graminoids and ericoids, in *Sphagnum-*dominated peatlands^8^. The enhanced root exudation associated with the vegetation shift could increase a role of positive priming effect in peatland C cycling^9^.

Exudate inputs to the soil commonly stimulate decomposition of pre-existing organic matter, causing a positive priming effect. However, a negative or no priming effect can also occur. A meta-analysis of data from terrestrial ecosystems^10^ showed that addition of simple organic compounds could cause both deceleration (down to 50%) and acceleration of SOC decomposition (up to 400%). Similarly, a modelled priming effect of simple organic compounds on recalcitrant DOC in aquatic ecosystems ranged from − 130 to + 370%^11^. Although the impact of easily decomposable compounds on decomposition significantly varies among systems, it can be generalized that a low input of easily decomposable compounds leads to negative or no priming effect, while the high labile C addition can induce strong positive priming^12^ and organic acids stimulate SOC decomposition more than carbohydrates^13^. Besides organic acids and carbohydrates, root exudates also contain nitrogen (N)^14^. C-rich root exudation stimulates microbial growth and enhances N and phosphorus (P) demand to build biomass, which may lead to enzymatic nutrient mining from SOC and DOC^15^. Accordingly, increasing positive PE was found with increasing C/N ratio of exudates^16^ and under soil N limitation compared to replete N conditions^17^. The phenomenon of nutrient mining from native DOC after the addition of C-rich exudates could play an important role in microbial metabolism within nutrient limited peatland ecosystems.

In peatlands, *Sphagnum* mosses are accompanied by vascular plants inhabiting microsites with lower water level. Peatland plant species differ in exudation flux and composition, and additionally, there is temporal variation in exudation as shown by Edwards et al.^18^. Comparing three peatland plant species, they found the highest exudation rate for *Eriophorum vaginatum*, lower for *Vaccinium myrtillus* and the lowest for *Sphagnum fallax.* Organic acids with much lower contribution of sugars and amino acids dominated the exudate pool. Exudate C/N ratios ranged from 8 to 80, with *Sphagnum* exudates being of higher C/N (lower quality) compared to *Eriophorum* and *Vaccinium* exudates, which contained more organic and inorganic N (higher quality). The exudate C/N ratio of all species increased from spring to autumn (i.e., decreased in quality over time). Root exudation of vascular plants contributed from 1 to 5% to the peatland DOC^18^. Studied plants by Edwards et al.^18^ are major plant dominants of spruce swamp forest located in the Šumava Mountains, in which our porewater samples were collected.

The root exudates entering the soil can influence the structure and function of the rhizosphere microbial community and cause successional changes, generally described in the mechanistic model of Andrews and Harris^19^. First root exudates stimulate the growth of r-strategists [i.e., early colonizers;^20^], commonly Gram-negative bacteria such as *Gammaproteobacteria* and *Bacteriodetes* but also fungi^21^. Due to fast growth, this population requires sufficient amounts of N and P, which are immobilized in its biomass^20^. After depletion of easily degradable compounds and available nutrients, r-strategists die and their products and necromass serve as a substrate for a developing K-strategic population (i.e., superior competitors) (e.g. *Acidobacteria* and *Planctomycetes*)^22^. To cleave and utilize such substrates, the K-strategists produce extracellular enzymes, which may lead to a targeted or co-metabolic decomposition of pre-existing, more stable SOC and finally result in positive priming effect^23^. The composition of microbial community actively utilizing the present substrates, its succession and potential contribution of particular bacterial and fungal representatives to priming effect depend not only on the quantity and quality of entering exudates but also on initial composition of microbial community^24^. Because of the prevailing microaerophilic or anaerobic nature of peatlands, bacteria dominate the microbial community^25^, while fungi are suppressed, and their abundance increases only in oxic microsites^26^. It is therefore likely that bacteria will play a dominant role in the decomposition of plant exudates, recalcitrant peatland DOC and in the priming effect, which may be induced by root exudate inputs.

In this study, we aimed to (1) evaluate the effect of the input and C/N ratio of root exudates on recalcitrant DOC decomposition with a focus on its dynamics and resulting priming effect, (2) identify the compositional and functional changes in the microbial community during exudate and DOC decomposition, and (3) identify microorganisms with high degradation potential which can be linked to the positive priming effect. To determine the dynamics of exudate and peatland DOC decomposition and priming effect quantification, we needed to separate these two C sources and monitor the system for a longer period. This would be impossible in the field due to heterogeneous conditions and DOC interaction with peat (sorption, chemical and microbial transformation) during porewater flow through the peat profile. We conducted a laboratory experiment in which we used original peat recalcitrant DOC enriched with microbial inoculum prepared from the peat, into which we added a mixture of ^13^C labelled organic compounds simulating root exudate inputs. The exudate addition differed in quantity (2% or 5% of present DOC) and in C/N stoichiometry (C/N ratios 7, 25, 50) simulating different qualities of root exudation of peatland plant species during the growing season (according to Edwards et al.^18^).

Based on the literature survey we hypothesized:

1. Addition of root exudates will stimulate decomposition of recalcitrant peatland DOC resulting in a positive priming effect. The positive priming effect will increase with increasing level of added exudates and their increasing C/N ratio.
2. Addition of root exudates to porewater DOC will lead to successional changes in the present microbial community: Initially, the presence of fresh exudates will increase the abundance of r-strategic species, while later the proportion of K-strategist will increase albeit the community will be smaller. Namely, K-strategic species with a larger degradation potential (potential to produce extracellular enzymes decomposing complex substrates) will be responsible for the positive priming effect.

## Results

### Total respiration rates and priming effect

**Figure 1:**
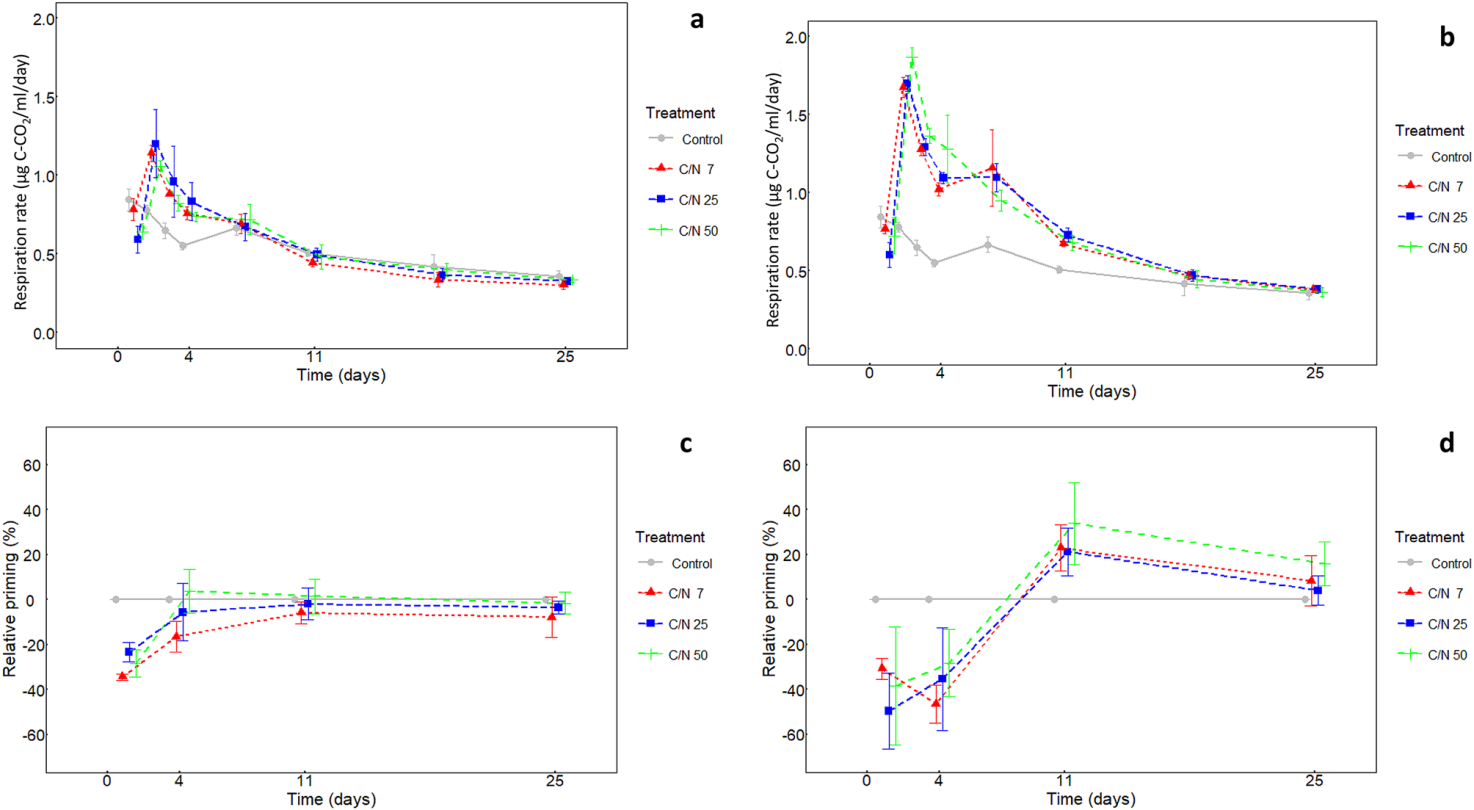
Respiration rate of control samples and samples amended by artificial exudates with different C/N ratios (**A**) in concentration of 2% of total DOC and (**B**) in concentration of 5% of total DOC during 25 days incubation. (**C**) Relative priming effect on DOC induced by artificial exudates with different C/N ratios in concentration of 2% of total DOC and (**D**) in concentration of 5% of total DOC during 25 days incubation (means, ± standard deviations, n = 4).

Samples with root exudate addition initially had lower total respiration rates than control (peatland porewater only) (Fig. 1a,b). However, from day 2, the enriched samples respired significantly more than control (*p* < 0.001). The stimulatory effect of exudate addition on total respiration rate was higher and lasted longer for 5% exudates level (Fig. 1b) than for 2% level (Fig. 1a). Specifically, samples with 2% exudate level respired more than control only until day 4 (*p* < 0.001) (Fig. 1a), while samples with 5% exudates level until day 11 (*p* < 0.001) (Fig. 1b). The exudate C/N ratio did not affect total respiration rate in any treatment and on any time. After 25 days of incubation, from 17.1 to 23.5% of total C (DOC + exudate C) were transformed to CO_2_.

The priming effect on DOC induced by exudate addition was dynamic in terms of time. The addition of exudates at both concentrations (2% and 5%) initially induced negative priming effect (Fig. 1c,d) until day 4, whereas between day 4 and 11, the priming effect turned to significantly positive for 5% exudate level (*p* < 0.001). The exudates with C/N ratio of 50 induced larger positive priming effect as compared to the exudates with lower C/N on the day 11 (*p* < 0.01) and only that one remained positive also on the day 25 (*p* < 0.05) (Fig. 1d). Differently, no priming effect or slightly negative priming effect was measured for 2% exudate addition until the end of experiment, with no effect of exudate C/N ratio on priming effect (Fig. 1c). The final priming effect ranged from − 7.9 to − 1.2% for low exudates level while the priming effect from 3.2 to 19.9% was measured for high exudates level. Significantly higher priming effect values were found for C/N ratio 50 than 7 for high level of exudates addition.

### Nutrient concentrations as affected by exudates addition

**Figure 2:**
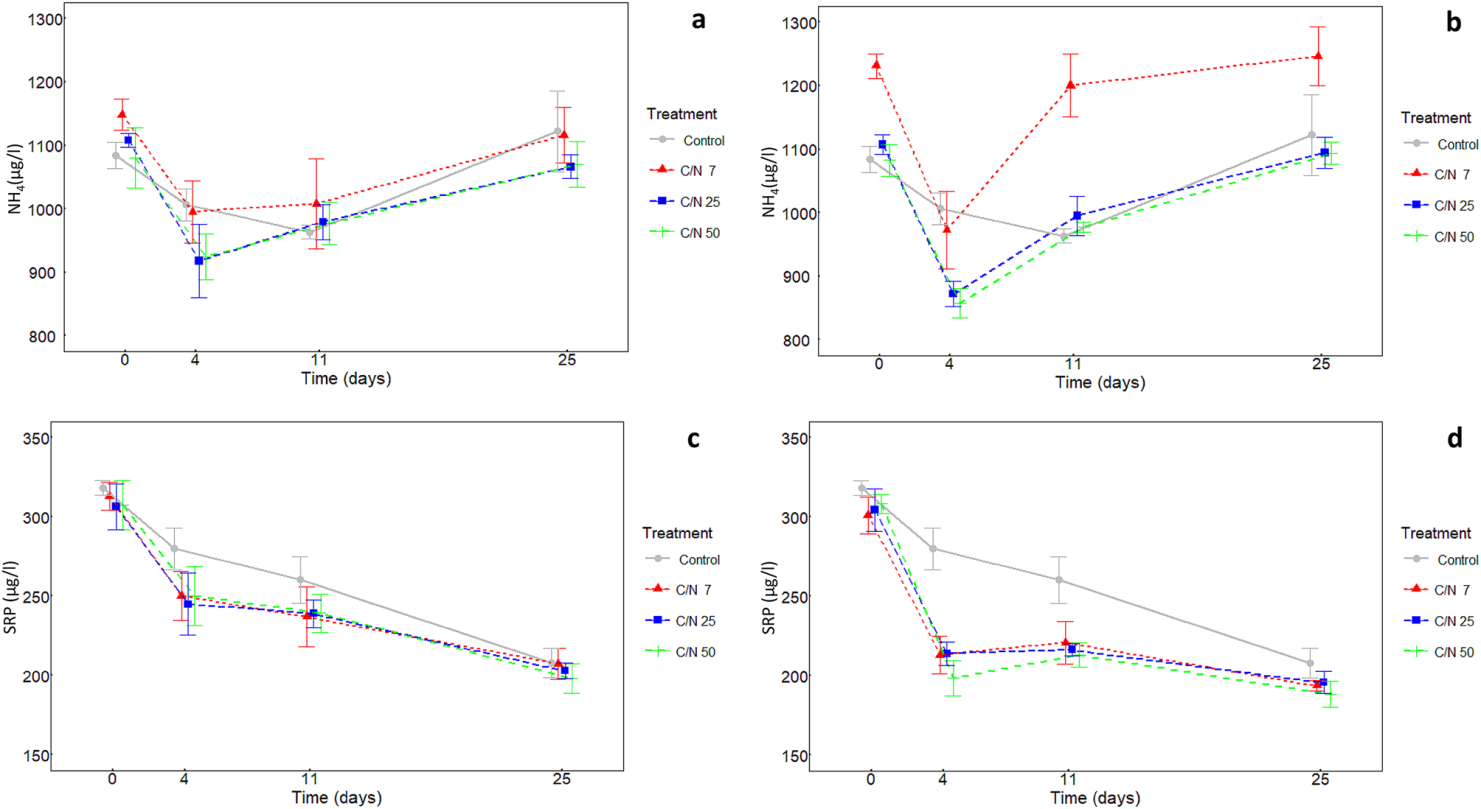
Ammonium N (NH_4_) concentration in control samples and samples amended by artificial exudates with different C/N ratios (**A**) in concentration of 2% of total DOC and (**B**) in concentration of 5% of total DOC during 25 days incubation. (**C**) Soluble reactive phosphorus (SRP) concentration in control samples and samples amended by artificial exudates with different C/N ratios in concentration of 2% of total DOC and (**D**) in concentration of 5% of total DOC during 25 days incubation (means, ± standard deviations, n = 4).

Concentration of ammonium N in the peatland porewater of samples enriched with exudates significantly decreased during first four days (except 2% exudates with C/N ratio 7) (*p* < 0.001) and then slowly increased back close to its original levels until day 25. Similar dynamics of ammonium N occurred also in the control but the changes were much less pronounced and the decrease lasted until day 11 (Fig. 2a,b). Differently, no significant changes were found for nitrate (Supplementary Fig. S1a,b). Soluble reactive phosphorus (SRP) concentration decreased in time (*p* < 0.001), with the fastest decline between day 0 and 4, followed by relatively stable phase between day 4–11 and another but slower decrease until the end of incubation (Fig. 2c,d). The decline of SRP in the solution was more pronounced under 5% than 2% level of exudates addition (*p* < 0.001). The SRP declined also in controls but the decline was stable, without an initial sharp stage. After 25 days of the incubation, samples with 5% exudates had still significantly lower SRP concentration than control, while those with 2% exudates did not differ from the control (*p* < 0.01).

### Temporal changes in abundance and composition of microbial communities

**Figure 3:**
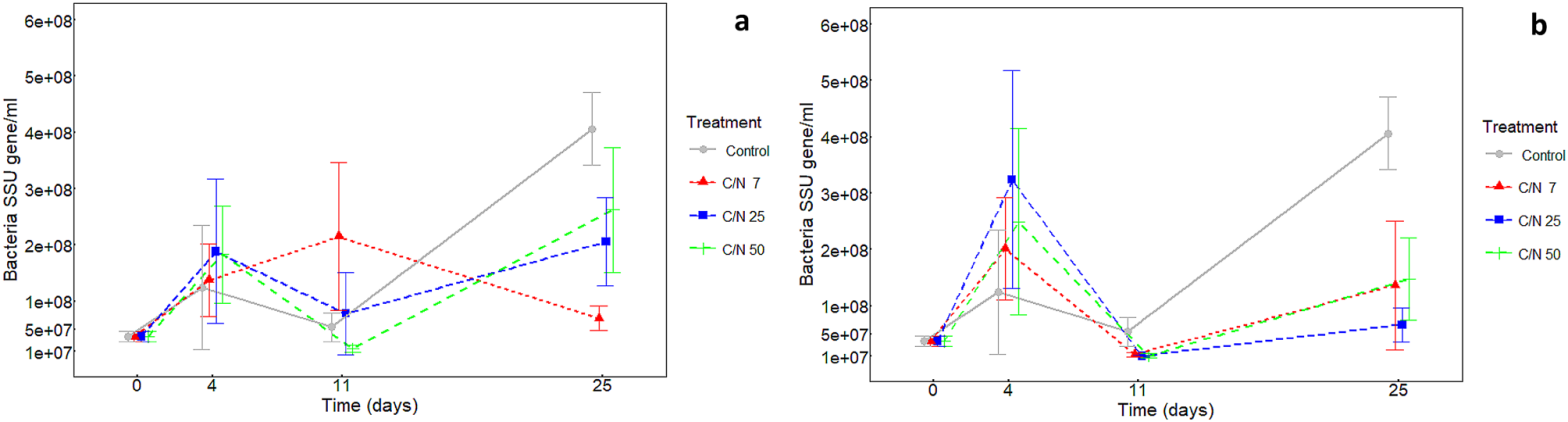
Bacteria SSU gene (per ml) in control samples and samples amended by artificial exudates with different C/N ratios (**A**) in concentration of 2% of total DOC and (**B**) in concentration of 5% of total DOC during 25 days incubation (means, ± standard deviations, n = 4).

Generally, the microbial community in peatland water was dominated by bacteria. Fungi formed a minor part of the microbial community, which is shown by an extremely low fungi to bacteria ratio (F/B ratio), which ranged from 1.15*10^−5^ to 0.026 (Supplementary Fig. S2a,b). Further, bacterial abundance was more dynamic than fungal abundance after exudate addition. The abundance of bacterial marker genes increased during the first 4 days of incubation across all treatments (Fig. 3). This growth was followed by a significant decrease until day 11 in the 5% level addition (Fig. 3b). In the 2% level addition, the bacterial abundance was similarly dynamic only for exudates with C/N 50, while bacterial abundance did not change significantly for other exudate C/N ratios (Fig. 3a). Fungal abundance increased from day 11 until day 25 and, therefore, the F/B ratio increased compare to original conditions (*p* < 0.05) (Supplementary Fig. S2). Finally, samples with both 2% and 5% exudate addition had higher F/B ratio than control and, additionally, those enriched with exudates with C/N ratio of 7 had higher F/B ratios than other treatments (*p* < 0.01).

Bacterial community composition significantly changed in time under both levels of exudate addition, being unique each sampling day. The temporal changes of bacterial community composition explained 31.7% of data variability in samples with 2% exudate addition and 59.7% of variability in treatment with 5% exudates (Supplementary Fig. S3).

**Figure 4:**
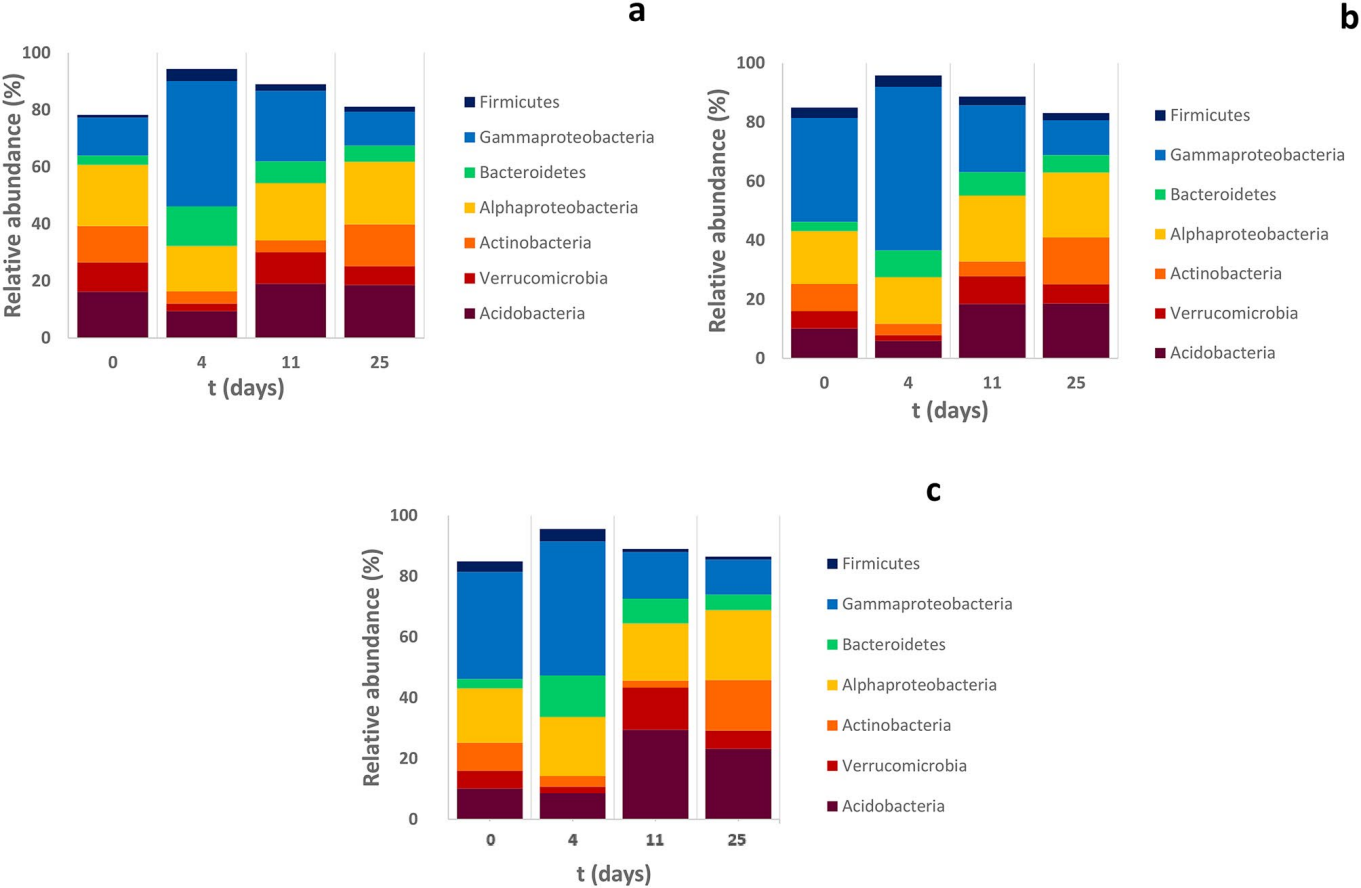
The relative abundance of the main phyla and classes in samples amended by artificial exudates in concentration of (**A**) 2% of total DOC, (**B**) 5% of total DOC and (**C**) control during 25 days incubation.

During the early stage of the decomposition, *Gammaproteobacteria* was the most abundant class*,* specifically at higher dose (5% treatment) of exudates (Fig. 4). This was caused by a significant increase in relative abundance of *Pseudomonas* (at both levels of exudate addition but more pronounced at 5% level) and *Burkholderia* (only at 5% level of exudate addition) (Table 1). Other genus from *Gammaproteobacteria, Masillia* responded oppositely and its proportion in the community decreased after exudate addition. *Bacteroidetes* phylum was enriched during early stage of the decomposition. In more detail, *Mucilaginibacter* genus proportion and abundance was increasing together with level of added root exudates (Table 1).

**Table 1:** Significant differences in relative abundance (%) of bacterial genera between the control and treatments with added root exudates (2% or 5%) at particular sampling times.

Time (day)	4		11		25	
Treatment (addition level)	2%	5%	2%	5%	2%	5%
**Acidobacteria**						
*Bryocella*	–	–	–	1.43	0.28	0.66
*Candidatus_Solibacter*	–	–	0.03	–	0.08	0.96
*Granulicella*	–	–	0.02	–	− 2.71	− 4.4
*Telmatobacter*	–	− 0.3	–	–	− 1.2	− 1.13
**Gammaproteobacteria**						
*Burkholderia*	–	20.55	–	–	–	0.08
*Legionella*	–	–	1.16	4.42	0.11	0.29
*Massilia*	− 5.63	− 7.41	–	− 0.38	–	–
*Pseudomonas*	5.56	10.95	0.33	0.49	0.02	0.02
**Bacteroidetes**						
*Mucilaginibacter*	0.79	2.67	0.21	0.51	0.1	0.08

At day 11, *Proteobacteria* were still more abundant and *Bacteriodetes* and *Acidobacteria* also increased their relative abundance in amended samples compare to control (Fig. 4). These higher abundances were mainly attributed to *Legionella* and *Pseudomonas* (*Proteobacteria*) and *Bryocella* (*Acidobacteriaceae* (*Subgroup* 1, Table 1)).

After 25 days of incubation, microbial communities of amended samples had significantly lower proportions of *Acidobacteria* as compared to control. However, looking deeper into the taxonomy of *Acidobacteria*, there were large differences in the response of specific genera. *Granulicella* and *Telmatobacter* (both belonging to *Acidobacteriaceae* (*Subgroup* 1)) decreased their proportion in the community, while *Candidatus Solibacter* (*Acidobacteria*, *Subgroup 3*) compred to control while *Bryocella* (Acidobacteriaceae (Subgroup 1)) was enriched in control (Table 1; for more details about bacterial and fungal community see Supplementary Tables S1 and S2, respectively).

We investigated a response of microbial community to 5% addition with the exudate C/N ratio 50 (Table S1) in the detail, because this addition induced the most pronounced changes in bacterial community and was connected with the largest positive priming effect (Fig. 1). In the early stage of decomposition, *Methylocella* was enriched in the treatment with high exudate C/N ratio up to 0.18% as compared to both C/N ratio 7 and 25 (*p* < 0.05; Supplementary Table S3). At day 11, the C/N ratio 50 had the highest increase of fungal genus *Rhodotorula* by 25.1% as compared to C/N ratio 7 (*p* < 0.05) and was enriched by 0.4% with *Methylobacterium* as compared to both C/N ratio 7 and 25. A significant positive response on exudates with C/N ratio 50 was found also for *Candidatus Solibacter* as compared to C/N ratio 7 and for *Legionella* and *Byssovorax* as compared to C/N ratio 25 by 0.5%, 1.5% and 0.3%, respectively (Supplementary Table S3).

### Temporal changes of functional potential of prokaryotic community

**Figure 5:**
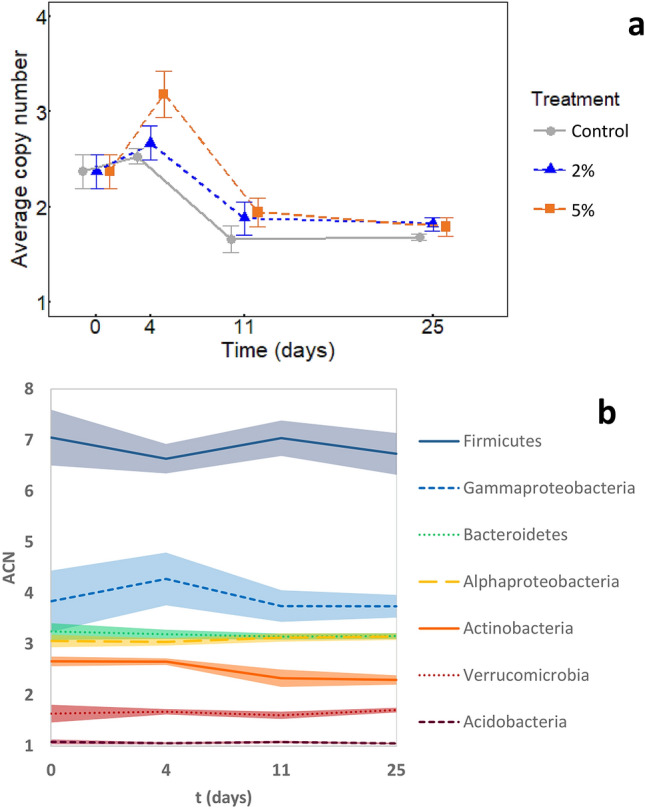
(**A**) r and K strategists in microbial community according to average 16rRNA copy number per genome (ACN) in control and samples amended by artificial exudates in concentration of 2% and 5% of total DOC during 25 days incubation. (**B**) Changes in each particular bacterial phylum in samples amended by artificial exudates in concentration of 2% of total DOC during 25 days incubation and (**C**) in concentration of 5% of total DOC during 25 days incubation. Enhanced ACN in the prokaryotic community indicates increased abundance of r-strategic species (means, ± standard deviations, n = 12).

Average 16SrRNA gene copy number per genome (ACN) can be used as the proxy for strategy shift of bacterial community, as increasing average ACN shows the higher proportion of the copiotrophic taxa^27,28^ while opposite is true for oligotrophic taxa. High level of exudates addition (5% treatment) caused a significant rise in ACN in the bacterial community mainly at the day 4 (Fig. 5), which indicates increased abundance of r-strategic species of gammaproteobacteria (Pseudomonas, Alcanindiges and Burkholderia). However, ACN decreased from day 4 to 11, but was still significantly higher than the control (*p* < 0.05). There was no change in ACN from 11 to 25 days and there were no differences among treatments. The ACN was negatively correlated with SRP concentration and positively correlated with respiration rate (r =  − 0.71 and r = 0.69, respectively; *p* < 0.05) at day 4 of the incubation. However, these correlations disappeared at day 11 and later (data not shown).

**Figure 6:**
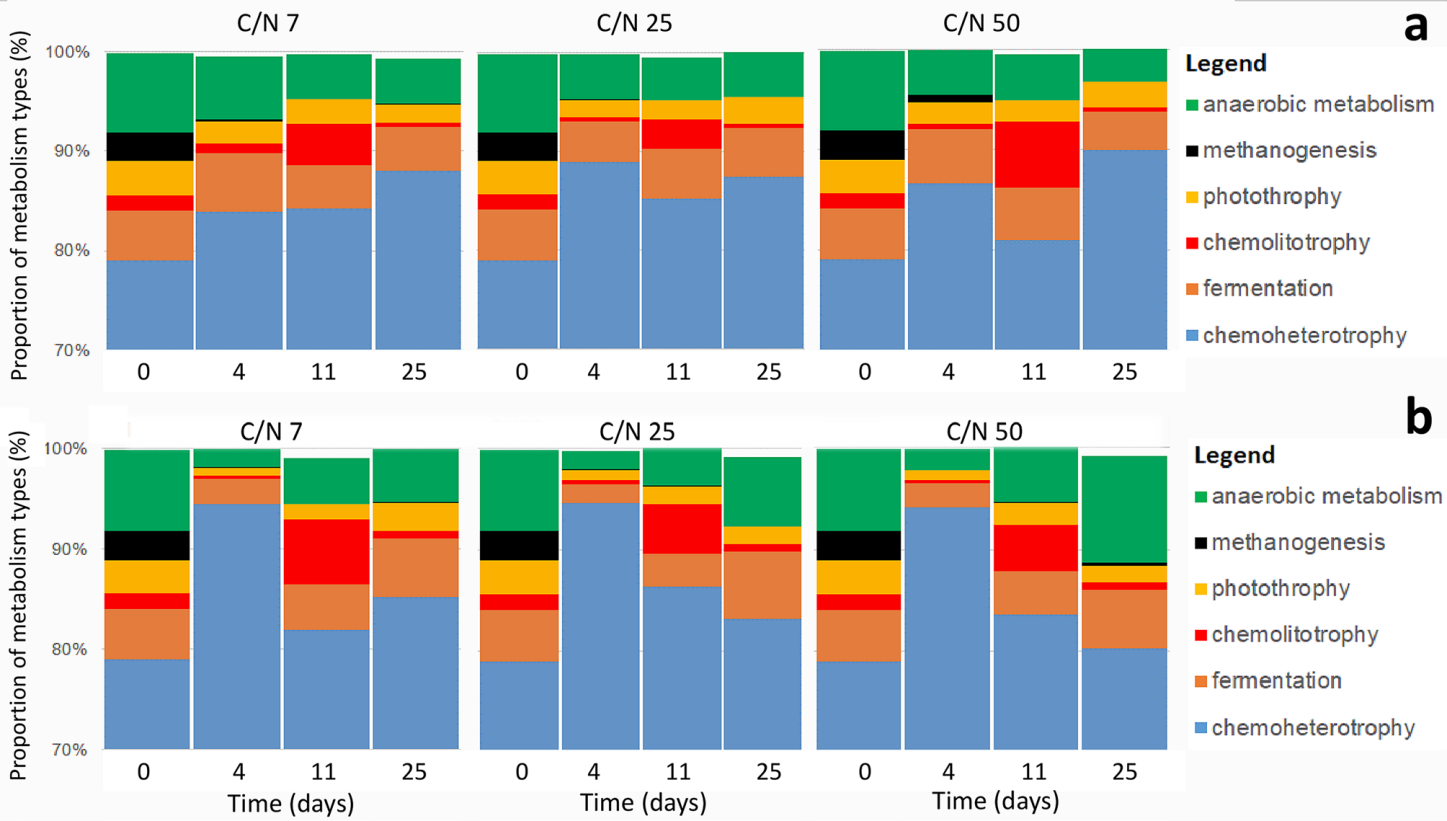
Temporal development of metabolism types of prokaryotic community in samples amended by artificial exudates with different C/N ratios (**A**) in concentration of 2% of total DOC and (**B**) in concentration of 5% of total DOC during 25 days incubation. Type of metabolism was assigned according to known bacterial genomes using FAPROTAX algorithm (Louca et al.^47^).

The bacterial community contained 4096 zOTU, from which 37% were assigned to metabolic or other ecologically relevant functions using FAPROTAX bioinformatic pipeline. The main metabolic guilds showed distinct temporal behavior after 2% and 5% exudate addition (Fig. 6). At day 4, the 5% level of exudates addition significantly (*p* < 0.05) increased the proportion of chemoheterotrophic prokaryotes while the proportion of anaerobes including fermenting bacteria decreased. This trend was almost identical for all exudates C/N ratios. Contrary, the day 11 was characteristic by a diminished chemoheterotrophy and a relative increase of chemolitotrophs across all exudate C/N ratios, from which 90% were ammonia oxidizers. Similar changes occurred also after 2% level of exudate addition but these changes were mostly not significant. The 5% level of exudates addition also increased the proportion of anaerobes at the end of incubation (25th day) at all C/N ratios but specifically at the highest C/N ratio (for more detailed statistical analysis of functional potential of prokaryotic community see Supplementary Table S4).

Functional analysis revealed that bacteria capable to decompose complex organic matter (*Pseudomonas, Burkholderia*) were significantly enriched during first four days in all treatments (including control).The increase was more pronounced after the 5% level of exudate addition, where these bacteria formed 6.5% OTU compared to 5.8% in both control and low level of exudate addition (*p* < 0.001). Afterwards, the bacteria able to decompose complex material increased in both amended treatments as compared to control by ca 0.4% in 11 days (control—4.7%) and by ca 0.2% at the end of incubation (control—5.2%).

## Discussion

### Priming effect was influenced by quantity and C/N ratio of added exudates

The resulting priming effect on peatland DOC decomposition was primarily affected by the exudate input: the low level of root exudates addition (2% of total DOC) to peatland water resulted in either a slightly negative (for C/N ratio 7) or no priming effect (for C/N ratio 25 and 50) on pre-existing peatland DOC (Fig. 1c), whereas the higher level of root exudates (5% of total DOC) induced a positive priming effect, which further increased with an increasing exudate C/N ratio from 7 to 50 (Fig. 1d). Our results are in line with findings of Liu et al.^13^ who found that low level of C addition led to a negative or no priming effect during a 7 week incubation, while the high level of C addition induced positive priming and of Wang et al.^29^, who observed an increasing priming effect with an increasing C/N ratio of crop residues added to the soil.

The priming effect was a dynamic process, closely related to the growth and succession of the present microbial community. The growth and the composition of the microbial communities in the samples were primarily controlled by the level of root exudates addition. While the low level of exudate addition enabled only limited microbial growth, the high exudate addition level resulted in a significant temporary increase in microbial abundance (especially bacterial abundance). Therefore, the related growth nutrient demand and a capacity to produce extracellular enzymes and decompose the native DOC were larger after the high exudate input. The rapidly growing communities first took up the available nutrients, as shown by a pronounced depletion of ammonia and SRP from the incubated solutions. Later, the decomposition of native DOC increased in samples enriched by high exudate level as a result of co-metabolism and/or a targeted nutrient mining^16^.

The two distinct phases of exudate and DOC utilization were connected with a significant successional development of bacterial community size (Fig. 3) and composition (Fig. 4), similarly as observed by Shi et al.^14^. The highest shift in the bacterial community composition occurred between 4 and 11th day but only in the 5% exudate addition treatment (Fig. 4). We compare the two stages (early stage and late stage) varying in exudate and DOC decomposition, microbial abundance and community composition in more detail below.

The early stage of incubation immediately following the exudate addition (until day 4) was characterized by a preferential use of the added compounds by the microbial community, resulting in a strong negative priming effect (Fig. 1). The exudate addition induced transitional bacterial growth connected with a rapid uptake of SRP and inorganic N from the solution and subsequent depletion of simple exudates from the solution. The r-strategic bacteria, mainly the representatives of *Gammaproteobacteria* and *Bacteriodete*s, enhanced their relative abundance within the communitycompared to the control (Fig. 4a–c). These are known as copiotrophic bacteria with a rapid turnover^27^, which are often stimulated by the presence of labile substrates like root exudates^30^. The greatest increase was observed for *Burkholderia, Pseudomonas* (*Proteobacteria*) and *Mucilaginibacter* (*Bacteroidetes*), which are typical by their large metabolic versatility^31^. *Pseudomonas* and *Burkholderia* are able to degrade complex aromatic compounds including lignin and other phenolics^30^. *Pseudomonas* belongs among the most efficient phosphate solubilizers due to production of organic acids^32^. Therefore, *Pseudomonas* could facilitate nutrients (mainly P) uptake by plants, especially in nutrient limited environment like peatlands.

In accordance with the observed community enrichment by the efficient decomposers, our functional analysis showed a large potential of the present bacterial community to degrade complex organic compounds, including cellulose and aromatics. We therefore suggest that this community dominated by the r-strategic taxa (e.g., *Gammaproteobacteria* and *Bacteriodete*s) (Fig. 5a) with a large decomposition potential largely contributes to the positive priming effect, which was observed at 11th day (Fig. 1). This partly contradicts a classical understanding of the priming effect mechanism, which suggests that the main role in production of exoenzymes degrading complex SOC is played by K-strategists, which follow in the succession, when the r-strategic community dies after a depletion of simple substrates^20^.

Exudate depletion from the solution occurred before day 4 as indicated by a sharp decrease in microbial respiration rates (Fig. 1) and was followed by a decline of bacterial abundance between days 4 and 11 mainly (Fig. 3).

4 and 11 mainly. For example *Bryocella* (Acidobacteria) which was more abundant at day was 11 was shown to have high enzymatic activity^31^ and thus may contribute to the significant positive PE observed at day 11 (Fig. 2). We also hypothetize that after exhaustion of the exudates, the r-strategists lost a competitive advantage over the K-strategists. Community probably shifted from short-term increase of r-strategists back to smaller K-strategic community composed *Alphaproteobacteria, Actinobacteria* and *Acidobacteria* (Fig. 4)*.* For example, members of *Acidobacteria* are efficient cellulose decomposers^31,33^ and their high abundance in microbial community may drive the litter degradation in acidic *Sphagnum* peat^33^. Another *Acidobacteria*, *Candidatus Solibacter,* was one of a few genera enriched under high C/N ratio at the end of the incubation (day 25). This genus is capable of cellulose, hemicellulose and chitin degradation and may contribute to the DOC decomposition in the later stage of incubation. We thus suggest that the enhanced DOC decomposition and the observed positive PE at days 11 and 25 can be attributed to a synergic of enzymes produced by both the r-strategists activated quickly right after exudate addition and by later slower growing K-strategists.

Except of the above mentioned heterotrophic bacteria, the community characteristic for the later stage of incubation, specifically for the 11th day, was further enriched in chemolithotrophic ammonia oxidizers (Fig. 6). Their larger presence was likely enabled by a depletion of simple organic substrates and a die-back of fast-growing bacteria, which could otherwise over compete these slow growing microbes. Moreover, the biomass die-back and the DOC decomposition enhanced the availability of NH_4_^+^, which is used in their energetic metabolism.

Edwards et al.^19^ estimated that root exudates of peatland vascular plants, which were easily degradable, could contribute from 1% up to 5% to the pre-existing peatland DOC in situ. Our results evoke that a lower input of root exudates, achieving around 2% of DOC has a significant effect on composition of rhizosphere microbial community, but is insufficient to induce a significant positive priming effect on recalcitrant peatland DOC. However, when the exudate input increased to the level of 5% of the present DOC, it may induce a transient positive priming effect lasting several days. With exudates poor in N (C/N ratio of 50), the induced positive priming may persist for more than two weeks and result in an enhanced decomposition of the pre-existing DOC. According to Edwards et al.^19^, the situation, when the exudation input is high enough, occurs at the time of maximum plant biomass especially in the presence of graminoid species such as *Eriophorum vaginatum*. However, the exudates at that time were relatively rich in N, therefore likely not causing significant peatland DOC losses. We suggest that the plants may rather benefit from the changes in the composition of microbial community, which the exudates induce in their rhizosphere. According to our results, root exudate input supports a growth of r-strategic species, which are able to immobilize high amounts of nutrients in their biomass, keep them in the vicinity of plant roots, protect them from losses with the leaching DOC and potentially release them for the plant uptake during their fast turnover. The rhizosphere community is enriched in species like *Pseudomonas,* which can mobilize P and others (e.g. *Burkholderia* and *Mucilaginibacter)* with high metabolic potential. The presence of microbial communities able to keep nutrients in the rhizosphere of peatland vascular plants is further supported by our previous results from the field. In Kaštovská et al.^34^ we showed that the soil microbial biomass associated with *Eriophorum vaginatum* and *Vaccinium myrtillus* immobilizes large amounts of N and P present in the system.

Differently from root exudates of vascular plants, *Sphagnum* “exudates” are not expected to cause a significant positive priming effect on DOC decomposition. Although *Sphagnum*-released compounds can contribute up to 20% of peatland DOC, they are of low degradability being only around 15%^19^. We expect that these would not stimulate a growth of specific bacterial communities and their enzymatic production. Additionally, the compounds leached from *Sphagnum* were shown to immobilize P by its incorporation to the high molecular weight complexes and by co-precipitation with metals^35^ and they are known by their antimicrobial effects^28^.

In accordance with the study of Basiliko et al.^7^, we suggest that an input of root exudates from vascular plants may induce a positive priming effect on organic matter decomposition in the peatlands, but its importance for C transformation and ecosystem C balance is likely minor under current conditions. Current level of root exudation rather helps vascular plants to keep nutrients immobilized in the microbial biomass with fast turnover in the vicinity of the roots and thus facilitates their survival in the nutrient limited environment. However, if ongoing climate changes will result in a significant spread of vascular plants over peatlands, a priming effect caused by the enhanced root exudation could lead to higher dynamics of C cycle in peatlands and larger C mineralization.

In conclusion: 1/Priming effect was affected by level of root exudate addition: Low level of root exudate addition (~ 2% of total DOC) to peatland water caused negative priming effect whereas positive priming effect can occur with increasing level of root exudates (~ 5% of total DOC). Increasing C/N ratio from 7 to 50 enhances priming effect. Growing microbial community was limited by nutrients and the C excess induced by the input of C-rich exudates led to increased “microbial nutrient mining” from peatland porewater (Fig. 2).

2/Bacteria played a more important role than fungi in our experiment as indicated by the extremely low ratio fungi/bacteria (lower than 0.005). After root exudate addition, r-strategic bacteria abundance increased and their growth was connected with an increased uptake of SRP and ammonium N from peatland water. High level of added root exudates stimulated growth of microbial functional groups with a potential to decompose complex compounds. Therefore, our experiment indicates that r-strategic bacteria (mainly *Gammaproteobacteria*) importantly contribute to a positive priming effect on peatland DOC decomposition. The positive priming effect found in the treatments with high level of exudate addition, could be caused by both the enzymes produced by K-strategic bacteria and by the enzymes produced by r-strategic bacteria during their growth on simple exudates.

3/Detailed analysis of the microbial community revealed several genera, which could cause or contribute to positive priming effect on peatland DOC; *Burkholderia*, *Pseudomonas* and *Mucilaginibacter* stimulated by root exudate input were the most important groups of r-strategic bacteria; later followed by K-strategists *Bryocella* and *Candidatus Solibacter.*

## Methods

### Peatland water collection and preparation

Peatland water originated from a spruce swamp forest located in the Šumava Mountains southwest Czech Republic (49°1′28.04″N, 13°32′32.14″E). The dominant plants there are spruce (*Picea abies*), cotton-grass (*Eriophorum vaginatum*), blueberry (*Vaccinium myrtillus*) and peat mosses *(Sphagnum* spp.). The peatland water was sampled from the upper rooted layer (0–20 cm) in small depression in the plot where all the dominant plants were present. For more detailed description of the site see Kastovska et al.^34^. Peatland water was collected in October 2015. The water was filtered through the low-protein-binding Express PLUS Polyethersulfone membrane (GPWP) with a 0.22 µm pore size (Merck Millipore Ltd., Ireland). The filtrate was analysed for DOC (60 mg L^−1^) on a LiquiTOC II (Elementar, Germany) and for pH (pH = 4).

### Experimental design of incubation

Peatland water was incubated in 120 ml glass NTS vials. The control treatment consisted of the 75 ml of the filtered peatland water with the final DOC concentration of 60 mg C L^−1^. In experimental treatments, the peatland water was enriched by a mixture of simple organic compounds with different molar C/N ratio simulating root exudates. The basic mixture of artificial root exudates with the molar C/N ratio of 50:1 consisted of acetic acid, glucose and glutamic acid (^13^C labelled compounds, 99 at % of ^13^C, Sigma Aldrich), which contributed to the total C in the mixture by 75%, 15% and 10%, respectively^18,36^. The molar C/N ratios of 25:1 and 7:1 were adjusted by an addition of ammonium nitrate to the basic mixture of organics. The artificial root exudates were added to the peatland water in two concentrations representing 2% and 5% of the recalcitrant DOC concentration in four replicates. These concentrations were selected because they represent the natural range in the proportion root exudates contribute to the DOC pool^18^. The volume of added artificial exudates was 0.5 ml. Then the samples were inoculated by 0.5 ml of unfiltered supernatant prepared from the peat sampled from rooted upper layer at the same locality as the peatland water (10 g of peat shaken in 100 ml of distilled water for 1 h at 20 °C, and centrifuged at 1000 g for 5 min). After inoculation the vials were air-tightly closed with rubber stoppers and incubated on the roll-and-roll shaker at 20 °C for 25 days. The flasks with the samples were placed on the roll-and-roll orbital shaker and mixed continuously during the whole incubation experiment to prevent any oxygen or redox gradient formation.

At time 0, 4, 11 and 25 days after start of the incubation the solution (10 ml) was taken from each sample, filtered through the low-protein-binding Express PLUS Polyethersulfone membrane (GPWP) with a 0.22 µm pore size (Merck Millipore Ltd., Ireland)) and immediately analysed for soluble reactive P (SRP), ammonium N and nitrate N colorimetrically on flow injection analyser (FIA Lachat QC8500, Lachat Instruments, USA). The filtrate was analysed for DOC on a LiquiTOC II (Elementar, Germany).

### Respiratory C losses from the samples and priming effect

The CO_2_ and O_2_ concentration in the headspace of each flask was measured at 1, 2, 3, 4, 7, 11, 18 and 25 days of incubation using a HP 6850 gas chromatograph (Agilent, USA). After each measurement, the flasks were opened, ventilated and closed again. The CO_2_ data were used to calculate respiration rates and cumulative respiratory losses for each treatment, which were used as a proxy for decomposition losses. During the whole incubation O_2_ concentration did not drop below 18% and the highest measured CO_2_ concentrations were not higher than 9500 ppm (less than 1%).

At days 1, 4, 11 and 25, isotopic composition of the evolved CO_2_ was analysed by Gasbench II (Finnigan, Germany) connected with IRMS Delta X Plus (Finnigen, Germany). To calculate the amount of CO_2_ derived from exudates and pre-existing DOC, the following mass balance equations based on atom % were used:

$${\text{C}}_{{\text{T}}} {\updelta }_{T} { } = { }C_{EX} {\updelta }_{EX} { } + { }C_{DOC} {\updelta }_{DOC} { }$$

where *C*_T_ (*C*_T_ = *C*_EX_ + *C*_DOC_) is the total amount of CO_2_ during the considered time interval, *δ*_T_ is the corresponding isotopic composition, *C*_EX_ is the amount of CO_2_ derived from the added exudates, *δ*_EX_ is its isotopic composition in the exudates, *C*_DOC_ is the amount of CO_2_ derived from DOC and *δ*_DOC_ is its isotopic composition. The primed DOC-derived CO_2_ is the difference between the *C*_DOC_ of exudate-amended samples and C-CO_2_ efflux from control samples (*C*_control_). The extent of priming effect was expressed as relative (%) to respiration rate of control at particular sampling time.

$$ {\text{Priming}}\;{\text{effect }}\left( {\text{\% }} \right) = 100 \times \left( {C_{{DOC\;{\text{in}}\;{\text{exudate}} - {\text{amended}}\;{\text{samples}}}} {-}C_{Control} } \right)/C_{Control} $$

### Nucleic acid extraction and quantification

10 ml of peatland water was filtered as previously described. Filters were kept frozen until nucleic acid (DNA) extraction. Filters were cut into the small pieces to fit bead-beating tube. DNA was extracted from the filters according to modified bead-beating protocol^37^. Total DNA was quantified fluorometrically using Quantus fluorometer (Promega, USA) with Quantus DNA Start-Up Kit. Quality of DNA was also verified by agarose gel electrophoresis.

### DNA sequencing and microbial community analyses

The aliquots of DNA extracts were sent to SEQme company (Czech republic) for the preparation of a library and sequencing using HiSeq2500 platform. The Earth Microbiome Project (EMP) protocol was used for library preparation with modified universal primers 515FB/806RB^38^ and ITS1F/ITS2^39^ for prokaryotic 16S rDNA and fungal ITS1 amplicons, respectively. Bacterial 16SrDNA raw pair-end reads (150 bp) were joined and quality filtered using USEARCH v. 10.0.240 to obtain reads of approx. 250 bp length^40^. The fungal ITS1 region was extracted from reads using ITSx algorithm^41^. Both 16S and ITS1 amplicons were trimmed to equal lengths. Bacterial and fungal unique reads were grouped to zero-radius OTUS (zOTUs) using an UNOISE 3.0 algorithm, which includes also the removal of potential chimeric sequences^40^. Taxonomic assignment of each bacterial and fungal zOTU was done using blast algorithm (e-value = 0.001) and curated ARB Silva 132 database^42^ and UNITE v 7.2^43^. Raw sequencing data were deposited in European Nucleotide Archive (ENA) under the study ID PRJEB29666.

### Quantification of prokaryotic and eukaryotic microbial community

Quantification of bacterialand fungal SSU rRNA genes was performed using the FastStart SybrGREEN Roche Supermix and Step One system (Life Technologies, USA). Each reaction mixture (20 µl) contained 2 µl DNA template (~ 1–2 ng DNA), 1 µl each primer (0.5 pmol µl^−1^ each, final concentration), 6 µl dH2O, 10 µl FastStart SybrGREEN Roche Supermix (Roche, France) and 1 µl BSA (Fermentas, 20 mg µl^−1^). Initial denaturation (3 min, 95 °C) was followed by 30 cycles of 30 s at 95 °C, 30 s at 62 °C (bacteria), 15 s at 72 °C, and completed by fluorescence data acquisition at 80 °C used for target quantification. Product specificity was confirmed by melting point analysis (52–95 °C with a plate read every 0.5 °C) and amplicon size was verified with agarose gel electrophoresis. Bacterial DNA standards consisted of a dilution series (ranging from 10^1^ to 10^9^ gene copies µl^−1^) of a known amount of purified PCR product obtained from genomic *Escherichia coli* ATCC 9637 DNA by using the SSU gene-specific primers 341F/534R^44^. R^2^ values for the standard curves were > 0.99. Slope values were > 3.37 giving an estimated amplification efficiency of > 93%. The qPCR conditions for fungal quantification were as follows: initial denaturation (10 min, 95 °C) followed by 40 cycles of 1 min at 95 °C, 1 min at 56 °C, 1 min at 72 °C, and completed by fluorescence data acquisition at 72 °C used for target quantification. Fungal DNA standards consisted of a dilution series (ranging from 10^1^ to 10^7^ gene copies µl^−1^) of a known amount of purified PCR product obtained from genomic *Aspergillus niger* DNA by using the SSU gene-specific primers nu-SSU-0817-5′ and nu-SSU1196-3′^45^. R^2^ values for the fungal standard curves were > 0.99. The slope was between − 3.32 and − 3.5 giving estimated amplification efficiency between 97 and 91%, respectively. Detection limits for the various assays (i.e. lowest standard concentration that is significantly different from the non-template controls) were less than 100 gene copies for each of the genes per assay. Samples, standards and non-template controls were run in duplicates.

### Analyses of metabolic potential of the prokaryotic community

Two independent pipelines (PICRUSt, FAPROTAX) were used for in-silico prediction of the functional potential of the prokaryotic community. For PICRUSt^46^ and FAPROTAX^47^ analyses the rarified OTU tables to 2000 sequences generated by Qiime 1.9.1 were used with taxonomic classification based on GreenGenes database ver. 13.05. In general, the PICRUSt pipeline first normalized each OTU abundances by small ribosomal subunit gene (SSU) copy variation in bacterial genomes based on the most similar taxa. The resulting normalized table was then used for OTU functional annotation using known bacterial and archaeal genomes^46^. From the normalized OTU table we were additionally able to calculate community average genome SSU copy number (ACN) in each sample^48^. The ACN was calculated from the raw and normalized OTU table (1st step in PICRUSt pipeline). SSU gene copies range from 1 to 15 in microbial genomes. Copiotrophic microbes are assumed to have more SSU gene copies in genome, therefore the higher average ACN shows the higher proportion of the copiotrophic taxa in the microbial community.

### Statistical analyses

The effects of exudates additions, their C/N ratios and time on each variable (respiration rate, priming effect, nutrient concentrations, bacterial and fungal abundance) were tested by full-factorial repeated–measures analysis of variance (ANOVA) tests (Statistica 12, Dell, USA) followed by post hoc comparison (unequal N HSD test). The significant differences (Welchs t-test, two-sided, Bonferroni correction) of phyla and genera between different treatments and control were analysed in STAMP v2.1.3^49^.

## Supplementary Information

Supplementary Information.

## Data Availability

All data generated or analysed during this study are included in this published article (and its Supplementary Information files). Raw sequence data were deposited in European Nucleotide Archive (ENA) under the Accession No. PRJEB36145.
