# Supplementary material for: Decomposition of peatland DOC affected by root exudates is driven by specific r and K strategic bacterial taxa
Source: Sci Rep. 2021 Sep 21;11:18677. doi: 10.1038/s41598-021-97698-2 (PMC8455546; doi:10.1038/s41598-021-97698-2)

Decomposition of peatland DOC affected by root exudates is driven by specific r and K strategic bacterial taxa

Jiří Mastný[1], Jiří Bárta[1], Eva Kaštovská[1], Tomáš Picek[1]

1 Department of Ecosystem Biology, Faculty of Science, University of South Bohemia, Branišovská 1760, České Budějovice 37005, Czech Republic

Corresponding author: Jiří Mastný; mastnj00@prf.jcu.cz

Supplementary:

Table S1: Changes in bacterial community after root exudates addition (2% or 5% of total C).

2 % of added root exudates											
4 days											
**Phylum**	**Class**	**Order**	**Family**	**Genus**	**OTUs**	**Control mean rel. freq. (%)**	**Control : std. dev. (%)**	**2%: mean rel. freq. (%)**	**2%: std. dev. (%)**	**p-value**	**Difference between means**
Proteobacteria	Betaproteobacteria	Burkholderiales	Oxalobacteraceae	**Undibacterium**	Zotu18	4.18	0.88	**6.59**	1.98	0.01	**2.41**
Proteobacteria	Gammaproteobacteria	Pseudomonadales	Pseudomonadaceae	**Pseudomonas**	Zotu8	1.36	0.42	**6.36**	2.92	0.00	**4.99**
Proteobacteria	Betaproteobacteria	Burkholderiales	Oxalobacteraceae	**Massilia**	Zotu3	10.08	2.99	**4.45**	1.04	0.04	**-5.63**
Firmicutes	Bacilli	Bacillales	Family_XII	**Exiguobacterium**	Zotu42	0.68	0.27	**1.79**	0.58	0.00	**1.11**
Bacteroidetes	Sphingobacteriia	Sphingobacteriales	Sphingobacteriaceae	**Mucilaginibacter**	Zotu172	0.33	0.09	**0.89**	0.30	0.00	**0.57**
Proteobacteria	Gammaproteobacteria	Pseudomonadales	Pseudomonadaceae	**Pseudomonas**	Zotu174	0.09	0.05	**0.65**	0.30	0.00	**0.56**
Proteobacteria	Gammaproteobacteria	Enterobacteriales	Enterobacteriaceae	**Klebsiella**	Zotu124	0.35	0.13	**0.65**	0.31	0.04	**0.30**
Proteobacteria	Gammaproteobacteria	Pseudomonadales	Moraxellaceae	**Acinetobacter**	Zotu131	0.29	0.04	**0.60**	0.21	0.00	**0.31**
Proteobacteria	Gammaproteobacteria	Aeromonadales	Aeromonadaceae	**Aeromonas**	Zotu204	0.08	0.06	**0.40**	0.27	0.01	**0.33**
Proteobacteria	Betaproteobacteria	Neisseriales	Neisseriaceae	**Paludibacterium**	Zotu376	0.10	0.06	**0.37**	0.20	0.00	**0.27**
Proteobacteria	Alphaproteobacteria	Rhodospirillales	Rhodospirillaceae	**Azospirillum**	Zotu6	0.08	0.08	**0.37**	0.29	0.02	**0.29**

Acidobacteria	Acidobacteria	Subgroup_13	Uncultured bacterium	**Uncultured bacterium**	Zotu771	0.16	0.07	**0.34**	0.20	0.05	**0.17**
Proteobacteria	Alphaproteobacteria	Sphingomonadales	Sphingomonadaceae	**Blastomonas**	Zotu231	0.01	0.02	**0.24**	0.16	0.00	**0.23**
Proteobacteria	Gammaproteobacteria	Pseudomonadales	Moraxellaceae	**Acinetobacter**	Zotu254	0.05	0.06	**0.22**	0.21	0.05	**0.17**
**11 Days**											
**Phylum**	**Class**	**Order**	**Family**	**Genus**	**OTUs**	**Control mean rel. freq. (%)**	**Control: std. dev. (%)**	**2%: mean rel. freq. (%)**	**2%: std. dev. (%)**	**p-value**	**Difference between means**
Proteobacteria	Gammaproteobacteria	Legionellales	Legionellaceae	**Legionella**	Zotu54	0.68	0.44	**1.80**	1.04	0.04	**1.12**
Verrucomicrobia	Opitutae	Opitutales	Opitutaceae	**Opitutus**	Zotu29	0.48	0.31	**1.50**	0.52	0.02	**1.02**
Bacteroidetes	Sphingobacteriia	Sphingobacteriales	Chitinophagaceae	**Sediminibacterium**	Zotu24	0.22	0.06	**1.01**	0.38	0.00	**0.79**
Proteobacteria	Alphaproteobacteria	Rhizobiales	Methylocystaceae	**uncultured**	Zotu20	0.38	0.05	**0.58**	0.24	0.03	**0.20**
Proteobacteria	Betaproteobacteria	Burkholderiales	Oxalobacteraceae	**Undibacterium**	Zotu18	0.27	0.08	**0.54**	0.35	0.05	**0.27**
Proteobacteria	Gammaproteobacteria	Pseudomonadales	Pseudomonadaceae	**Pseudomonas**	Zotu8	0.13	0.08	**0.43**	0.19	0.01	**0.29**
Firmicutes	Bacilli	Bacillales	Family_XII	**Exiguobacterium**	Zotu126	0.07	0.09	**0.35**	0.34	0.04	**0.28**
Bacteroidetes	Sphingobacteriia	Sphingobacteriales	Sphingobacteriaceae	**Mucilaginibacter**	Zotu184	0.07	0.06	**0.34**	0.21	0.01	**0.27**
Armatimonadetes	Chthonomonadetes	Chthonomonadales	Chthonomonadaceae	**Unclassified Chthonomonadaceae**	Zotu109	0.13	0.02	**0.32**	0.13	0.00	**0.19**
Bacteroidetes	Sphingobacteriia	Sphingobacteriales	Chitinophagaceae	**uncultured**	Zotu87	0.12	0.06	**0.29**	0.15	0.03	**0.18**
Proteobacteria	Alphaproteobacteria	Rhizobiales	Methylobacteriaceae	**Methylobacterium**	Zotu141	0.03	0.02	**0.17**	0.15	0.01	**0.14**
Proteobacteria	Alphaproteobacteria	Rhodospirillales	Acetobacteraceae	**Acidocella**	Zotu389	0.02	0.02	**0.16**	0.11	0.00	**0.14**
Proteobacteria	Betaproteobacteria	Nitrosomonadales	Gallionellaceae	**Candidatus Nitrotoga**	Zotu393	0.02	0.02	**0.16**	0.12	0.00	**0.14**
Proteobacteria	Alphaproteobacteria	Rhodospirillales	Acetobacteraceae	**Unclassified Acetobacteraceae**	Zotu90	0.05	0.04	**0.16**	0.10	0.04	**0.11**
**25 Days**											

Phylum	Class	Order	Family	Genus	OTUs	Control mean rel. freq. (%)	Control : std. dev. (%)	2%: mean rel. freq. (%)	2%: std. dev. (%)	p-value	Difference between means
Acidobacteria	Acidobacteria	Acidobacteriales	Acidobacteriaceae_(Subgroup_1)	**Granulicella**	Zotu5	9.13	0.63	**6.40**	2.14	0.00	**-2.73**
Proteobacteria	Alphaproteobacteria	Rhizobiales	Xanthobacteraceae	**uncultured**	Zotu16	1.95	0.50	**3.15**	1.53	0.05	**1.20**
Verrucomicrobia	Spartobacteria	Chthoniobacterales	Chthoniobacteraceae	**Chthoniobacter**	Zotu9	0.83	0.41	**1.76**	0.79	0.02	**0.93**
Firmicutes	Bacilli	Bacillales	Family_XII	**Exiguobacterium**	Zotu42	0.46	0.19	**1.03**	0.62	0.02	**0.57**
Proteobacteria	Alphaproteobacteria	Rhodospirillales	Acetobacteraceae	**Unclassified Acetobacteraceae**	Zotu4	3.26	0.91	**1.20**	0.57	0.02	**-2.07**
Proteobacteria	Alphaproteobacteria	Rhodospirillales	Acetobacteraceae	**Unclassified Acetobacteraceae**	Zotu90	0.16	0.11	**0.51**	0.36	0.02	**0.35**
Acidobacteria	Acidobacteria	Acidobacteriales	Acidobacteriaceae_(Subgroup_1)	**Bryocella**	Zotu58	0.08	0.06	**0.36**	0.31	0.01	**0.28**
Acidobacteria	Acidobacteria	Acidobacteriales	Acidobacteriaceae_(Subgroup_1)	**Telmatobacter**	Zotu75	2.13	0.26	**0.93**	0.31	0.00	**-1.20**
Firmicutes	Bacilli	Bacillales	Family_XII	**Exiguobacterium**	Zotu126	0.16	0.04	**0.35**	0.25	0.04	**0.18**
Proteobacteria	Gammaproteobacteria	Pseudomonadales	Moraxellaceae	**Acinetobacter**	Zotu131	0.08	0.04	**0.25**	0.22	0.03	**0.18**
Proteobacteria	Alphaproteobacteria	Rhodospirillales	DA111	**Unclassified DA111**	Zotu103	1.25	0.38	**0.41**	0.21	0.03	**-0.84**
Proteobacteria	Deltaproteobacteria	Myxococcales	Polyangiaceae	**Byssovorax**	Zotu185	0.06	0.04	**0.22**	0.18	0.02	**0.16**
Proteobacteria	Gammaproteobacteria	Xanthomonadales	Nevskiaceae	**Alkanibacter**	Zotu377	0.01	0.02	**0.16**	0.18	0.02	**0.15**
Proteobacteria	Alphaproteobacteria	Sphingomonadales	Sphingomonadaceae	**Sphingomonas**	Zotu542	0.00	0.00	**0.11**	0.15	0.04	**0.11**
Proteobacteria	Alphaproteobacteria	Rhizobiales	Bradyrhizobiaceae	**Bradyrhizobium**	Zotu81	0.08	0.06	**0.25**	0.15	0.01	**0.18**
Planctomycetes	Planctomycetacia	Planctomycetales	Planctomycetaceae	**Singulisphaera**	Zotu256	0.03	0.04	**0.18**	0.15	0.01	**0.15**

**5 % of added root exudates**											
**4 Days**											
**Phylum**	**Class**	**Order**	**Family**	**Genus**	**OTUs**	**Control mean rel. freq. (%)**	**Control: std. dev. (%)**	**5%: mean rel.**	**5%: std.**	**p-value**	**Difference between means**

								freq. (%)	dev. (%)		
Proteobacteria	Betaproteobacteria	Burkholderiales	Burkholderiaceae	**Burkholderia**	Zotu1	8.30	5.98	**28.85**	6.58	0.00	**20.55**
Proteobacteria	Gammaproteobacteria	Pseudomonadales	Pseudomonadaceae	**Pseudomonas**	Zotu8	1.36	0.42	**11.80**	6.70	0.00	**10.44**
Bacteroidetes	Sphingobacteriia	Sphingobacteriales	Sphingobacteriaceae	**Mucilaginibacter**	Zotu145	0.13	0.08	**0.79**	0.60	0.00	**0.67**
Bacteroidetes	Sphingobacteriia	Sphingobacteriales	Sphingobacteriaceae	**Mucilaginibacter**	Zotu172	0.33	0.09	**0.84**	0.45	0.00	**0.51**
Proteobacteria	Alphaproteobacteria	Rhodospirillales	Acetobacteraceae	**Unclassified Acetobacteraceae**	Zotu4	8.21	0.27	**6.45**	1.59	0.00	**-1.76**
Proteobacteria	Betaproteobacteria	Burkholderiales	Oxalobacteraceae	**Massilia**	Zotu3	10.08	2.99	**3.07**	1.85	0.02	**-7.00**
Bacteroidetes	Sphingobacteriia	Sphingobacteriales	Sphingobacteriaceae	**Mucilaginibacter**	Zotu260	0.06	0.02	**0.42**	0.21	0.00	**0.36**
Proteobacteria	Betaproteobacteria	Burkholderiales	Oxalobacteraceae	**Undibacterium**	Zotu18	4.18	0.88	**1.19**	0.56	0.01	**-2.98**
Proteobacteria	Alphaproteobacteria	Sphingomonadales	Sphingomonadaceae	**Sphingomonas**	Zotu25	1.55	0.21	**1.04**	0.37	0.01	**-0.51**
Bacteroidetes	Sphingobacteriia	Sphingobacteriales	Sphingobacteriaceae	**Mucilaginibacter**	Zotu600	0.01	0.02	**0.08**	0.07	0.01	**0.07**
Firmicutes	Bacilli	Bacillales	Planococcaceae	**Planomicrobium**	Zotu234	0.01	0.02	**0.07**	0.05	0.02	**0.05**
Proteobacteria	Alphaproteobacteria	Rhizobiales	alphaI_cluster	**uncultured_bacterium**	Zotu50	1.53	0.51	**0.58**	0.24	0.05	**-0.94**
Proteobacteria	Gammaproteobacteria	Pseudomonadales	Pseudomonadaceae	**Pseudomonas**	Zotu174	0.09	0.05	**0.56**	0.40	0.00	**0.47**
Proteobacteria	Alphaproteobacteria	Rhizobiales	Methylobacteriaceae	**Methylobacterium**	Zotu141	0.11	0.09	**0.50**	0.22	0.00	**0.39**
Actinobacteria	Actinobacteria	Frankiales	Sporichthyaceae	**hgcI_clade**	Zotu1344	0.00	0.00	**0.04**	0.05	0.02	**0.04**
Acidobacteria	Acidobacteria	Subgroup_2	uncultured_bacterium	**uncultured_bacterium**	Zotu438	0.00	0.00	**0.04**	0.05	0.03	**0.04**
Proteobacteria	Betaproteobacteria	Burkholderiales	Oxalobacteraceae	**Massilia**	Zotu163	0.63	0.21	**0.22**	0.07	0.04	**-0.40**
**11 Days**											
**Phylum**	**Class**	**Order**	**Family**	**Genus**	**OTUs**	**Control mean rel. freq. (%)**	**Control: std. dev. (%)**	**5%: mean rel. freq. (%)**	**5%: std. dev. (%)**	**p-values (corrected)**	**Difference between means**
Verrucomicrobia	Spartobacteria	Chthoniobacterales	Chthoniobacteraceae	**Chthoniobacter**	Zotu9	10.97	2.11	**5.00**	2.18	0.04	**-5.96**
Proteobacteria	Gammaproteobacteria	Legionellales	Legionellaceae	**Legionella**	Zotu54	0.68	0.44	**3.07**	1.18	0.00	**2.39**

Proteobacteria	Gammaproteobacteria	Legionellales	Legionellaceae	**Legionella**	Zotu63	0.37	0.10	**2.28**	1.77	0.00	**1.91**
Verrucomicrobia	Opitutae	Opitutales	Opitutaceae	**Opitutus**	Zotu29	0.48	0.31	**2.08**	1.19	0.00	**1.59**
Bacteroidetes	Sphingobacteriia	Sphingobacteriales	Chitinophagaceae	**Sediminibacterium**	Zotu24	0.22	0.06	**1.91**	1.53	0.00	**1.69**
Proteobacteria	Alphaproteobacteria	Rhizobiales	Rhizobiales_Incertae_Sedis	**Rhizomicrobium**	Zotu22	0.92	0.16	**1.60**	0.39	0.00	**0.68**
Acidobacteria	Acidobacteria	Acidobacteriales	Acidobacteriaceae_(Subgroup_1)	**Bryocella**	Zotu58	0.10	0.11	**1.53**	1.44	0.01	**1.43**
Chlamydiae	Chlamydiae	Chlamydiales	Simkaniaceae	**Candidatus_Rhabdochlamydia**	Zotu13	0.17	0.13	**1.02**	1.14	0.03	**0.85**
Proteobacteria	Alphaproteobacteria	Rhodospirillales	Acetobacteraceae	**Acidisphaera**	Zotu82	0.40	0.18	**0.96**	0.53	0.02	**0.56**
Proteobacteria	Alphaproteobacteria	Rhizobiales	Methylocystaceae	**uncultured**	Zotu20	0.38	0.05	**0.96**	0.50	0.00	**0.58**
Proteobacteria	Alphaproteobacteria	Rhizobiales	Beijerinckiaceae	**uncultured**	Zotu30	0.45	0.11	**0.87**	0.40	0.01	**0.42**
Armatimonadetes	uncultured_Armatimonadetes_bacterium	uncultured_Armatimonadetes_bacterium	uncultured_Armatimonadetes_bacterium	**uncultured_Armatimonadetes_bacterium**	Zotu52	1.68	0.25	**0.86**	0.48	0.01	**-0.82**
Proteobacteria	Alphaproteobacteria	Rhizobiales	Beijerinckiaceae	**Methylocella**	Zotu76	0.17	0.14	**0.67**	0.38	0.01	**0.50**
Proteobacteria	Betaproteobacteria	Burkholderiales	Oxalobacteraceae	**Massilia**	Zotu3	1.23	0.21	**0.59**	0.25	0.03	**-0.65**
Proteobacteria	Alphaproteobacteria	Sphingomonadales	Sphingomonadaceae	**Sphingomonas**	Zotu25	1.02	0.14	**0.58**	0.25	0.02	**-0.44**
**25 Days**											
**Phylum**	**Class**	**Order**	**Family**	**Genus**	**OTUs**	**Control mean rel. freq. (%)**	**Control: std. dev. (%)**	**5%: mean rel. freq. (%)**	**5%: std. dev. (%)**	**p-values (corrected)**	**Difference between means**
Acidobacteria	Acidobacteria	Acidobacteriales	Acidobacteriaceae_(Subgroup_1)	**Granulicella**	Zotu5	9.13	0.63	**4.72**	1.65	0.00	**-4.40**
WCHB1-60	uncultured_bacterium	uncultured_bacterium	Unclassified uncultured_bacterium	**Unclassified uncultured_bacterium**	Zotu62	0.16	0.13	**2.05**	2.00	0.01	**1.88**
Bacteroidetes	Sphingobacteriia	Sphingobacteriales	Chitinophagaceae	**uncultured**	Zotu17	0.94	0.44	**1.95**	1.25	0.04	**1.01**
Verrucomicrobia	Opitutae	Opitutales	Opitutaceae	**Opitutus**	Zotu29	0.50	0.18	**1.55**	0.93	0.00	**1.05**
Acidobacteria	Acidobacteria	Subgroup_3	Unknown_Family	**Candidatus_Solibacter**	Zotu41	1.01	0.13	**1.51**	0.48	0.01	**0.50**

Proteobacteria	Alphaproteobacteria	Rhodospirillales	Acetobacteraceae	**Unclassified Acetobacteraceae**	Zotu4	3.26	0.91	**1.50**	1.66	0.04	**-1.76**
Proteobacteria	Alphaproteobacteria	Rhodospirillales	Rhodospirillales_Incertae_Sedis	**Reyranella**	Zotu45	0.35	0.22	**1.31**	0.72	0.00	**0.96**
Acidobacteria	Acidobacteria	Subgroup_3	Unknown_Family	**Candidatus_Solibacter**	Zotu57	0.56	0.25	**1.01**	0.40	0.04	**0.45**
Acidobacteria	Acidobacteria	Acidobacteriales	Acidobacteriaceae_(Subgroup_1)	**Telmatobacter**	Zotu75	2.13	0.26	**1.00**	0.44	0.00	**-1.13**
Acidobacteria	Acidobacteria	Acidobacteriales	Acidobacteriaceae_(Subgroup_1)	**Bryocella**	Zotu58	0.08	0.06	**0.73**	0.68	0.01	**0.66**
Bacteroidetes	Sphingobacteriia	Sphingobacteriales	CWT_CU03-E12	**uncultured_bacterium**	Zotu94	0.21	0.12	**0.52**	0.35	0.03	**0.30**
Firmicutes	Bacilli	Bacillales	Family_XII	**Exiguobacterium**	Zotu126	0.16	0.04	**0.35**	0.18	0.01	**0.18**
Proteobacteria	Alphaproteobacteria	Rhodospirillales	DA111	**Unclassified DA111**	Zotu103	1.25	0.38	**0.34**	0.14	0.02	**-0.91**
Proteobacteria	Gammaproteobacteria	Legionellales	Legionellaceae	**Legionella**	Zotu63	0.11	0.02	**0.29**	0.15	0.00	**0.18**
Proteobacteria	Gammaproteobacteria	Pseudomonadales	Moraxellaceae	**Acinetobacter**	Zotu131	0.08	0.04	**0.23**	0.22	0.05	**0.15**

Table S2: Changes in fungal community after root exudates addition (2% or 5% of total C).

2 % of added root exudates									
**4 days**									
**Phylum**	**Class**	**Order**	**Family**	**Genus**	**OTU**	**p-values (corrected)**	**Effect size**	**4: mean rel. freq. (%)**	**4: std. dev. (%)**
Ascomycota	Dothideomycetes	Dothideales	Saccotheciaceae	Aureobasidium	Otu3	**0.01**	0.25	31.39	33.21
Basidiomycota	Microbotryomycetes	Sporidiobolales	Sporidiobolaceae	Rhodotorula	Otu650	**0.00**	0.45	27.25	21.23
Basidiomycota	Microbotryomycetes	Sporidiobolales	Sporidiobolaceae	Rhodotorula	Otu1	**0.00**	0.58	3.31	2.06
Basidiomycota	Microbotryomycetes	Sporidiobolales	Sporidiobolaceae	Rhodotorula	Otu17	**0.00**	0.33	2.44	2.54
Ascomycota	Dothideomycetes	Dothideales	Saccotheciaceae	Aureobasidium	Otu1172	**0.07**	0.16	1.54	2.65
**11 days**									
**Phylum**	**Class**	**Order**	**Family**	**Genus**	**OTU**	**p-values (corrected)**	**Effect size**	**11: mean rel. freq. (%)**	**11: std. dev. (%)**
Basidiomycota	Microbotryomycetes	Sporidiobolales	Sporidiobolaceae	Rhodotorula	Otu650	**0.00**	0.45	41.07	16.60
Ascomycota	Dothideomycetes	Dothideales	Saccotheciaceae	Aureobasidium	Otu3	**0.01**	0.25	11.82	17.80
Basidiomycota	Microbotryomycetes	Sporidiobolales	Sporidiobolaceae	Rhodotorula	Otu1	**0.00**	0.58	4.66	1.48
Basidiomycota	Microbotryomycetes	Sporidiobolales	Sporidiobolaceae	Rhodotorula	Otu17	**0.00**	0.33	4.00	2.44
Ascomycota	Eurotiomycetes	Eurotiales	Aspergillaceae	Penicillium	Otu5	**0.07**	0.16	1.75	1.40
Ascomycota	Eurotiomycetes	Eurotiales	Aspergillaceae	Aspergillus	Otu51	**0.05**	0.17	1.70	2.66

Ascomycota	Geoglossomycetes	Geoglossales	Geoglossaceae	Sarcoleotia	Otu341	**0.01**	0.24	1.34	1.76
**25 days**									
**Phylum**	**Class**	**Order**	**Family**	**Genus**	**OTU**	**p-values (corrected)**	**Effect size**	**25: mean rel. freq. (%)**	**25: std. dev. (%)**
Basidiomycota	Microbotryomycetes	Sporidiobolales	Sporidiobolaceae	Rhodotorula	Otu650	**0.00**	0.45	40.70	14.10
Ascomycota	Dothideomycetes	Dothideales	Saccotheciaceae	Aureobasidium	Otu3	**0.01**	0.25	7.41	6.01
Basidiomycota	Microbotryomycetes	Sporidiobolales	Sporidiobolaceae	Rhodotorula	Otu1	**0.00**	0.58	5.64	1.62
Basidiomycota	Microbotryomycetes	Sporidiobolales	Sporidiobolaceae	Rhodotorula	Otu17	**0.00**	0.33	3.77	1.79
Ascomycota	Saccharomycetes	Saccharomycetales	Unclassified Saccharomycetales	Nadsonia	Otu438	**0.04**	0.18	1.24	1.90

**5 % of added root exudates**									
**4 day**									
**Phylum**	**Class**	**Order**	**Family**	**Genus**	**OTU**	**p-values (corrected)**	**Effect size**	**4: mean rel. freq. (%)**	**4: std. dev. (%)**
Basidiomycota	Microbotryomycetes	Sporidiobolales	Sporidiobolaceae	Rhodotorula	Otu650	**0.00**	0.41	39.87	22.35
Ascomycota	Dothideomycetes	Dothideales	Saccotheciaceae	Aureobasidium	Otu3	**0.02**	0.21	24.82	28.83
Basidiomycota	Microbotryomycetes	Sporidiobolales	Sporidiobolaceae	Rhodotorula	Otu1	**0.00**	0.63	4.50	2.06
Basidiomycota	Microbotryomycetes	Sporidiobolales	Sporidiobolaceae	Rhodotorula	Otu17	**0.01**	0.23	2.11	1.27
Basidiomycota	Tremellomycetes	Filobasidiales	Filobasidiaceae	Naganishia	Otu24	**0.00**	0.28	1.21	0.76
**11 day**									
**Phylum**	**Class**	**Order**	**Family**	**Genus**	**OTU**	**p-values (corrected)**	**Effect size**	**11: mean rel. freq. (%)**	**11: std. dev. (%)**
Basidiomycota	Microbotryomycetes	Sporidiobolales	Sporidiobolaceae	Rhodotorula	Otu650	**0.00**	0.41	41.66	16.36
Ascomycota	Dothideomycetes	Dothideales	Saccotheciaceae	Aureobasidium	Otu3	**0.02**	0.21	18.07	20.60
Basidiomycota	Microbotryomycetes	Sporidiobolales	Sporidiobolaceae	Rhodotorula	Otu1	**0.00**	0.63	4.60	1.91
Ascomycota	Sordariomycetes	Hypocreales	Clavicipitaceae	Metarhizium	Otu14	**0.02**	0.23	2.79	2.35
Basidiomycota	Tremellomycetes	Filobasidiales	Filobasidiaceae	Naganishia	Otu24	**0.00**	0.28	2.72	2.53
Ascomycota	Eurotiomycetes	Eurotiales	Trichocomaceae	Talaromyces	Otu519	**0.00**	0.41	41.66	16.36
Basidiomycota	Microbotryomycetes	Sporidiobolales	Sporidiobolaceae	Rhodotorula	Otu17	**0.02**	0.21	18.07	20.60
**25 day**									

Phylum	Class	Order	Family	Genus	OTU	p-values (corrected)	Effect size	25: mean rel. freq. (%)	25: std. dev. (%)
Basidiomycota	Microbotryomycetes	Sporidiobolales	Sporidiobolaceae	Rhodotorula	Otu650	**0.00**	0.41	37.77	18.42
Basidiomycota	Microbotryomycetes	Sporidiobolales	Sporidiobolaceae	Rhodotorula	Otu1	**0.00**	0.63	11.07	4.64
Ascomycota	Dothideomycetes	Dothideales	Saccotheciaceae	Aureobasidium	Otu3	**0.02**	0.21	4.57	4.53
Ascomycota	Sordariomycetes	Hypocreales	Clavicipitaceae	Metarhizium	Otu14	**0.02**	0.23	1.88	2.54
Basidiomycota	Microbotryomycetes	Sporidiobolales	Sporidiobolaceae	Rhodotorula	Otu17	**0.01**	0.23	1.87	1.33
Ascomycota	Eurotiomycetes	Eurotiales	Aspergillaceae	Penicillium	Otu495	**0.00**	0.41	37.77	18.42
Basidiomycota	Tremellomycetes	Filobasidiales	Filobasidiaceae	Naganishia	Otu24	**0.00**	0.63	11.07	4.64

Table S3: Changes in microbial community after addition of exudates with different C/N ratio(7, 25, 50)

Time	OTU/zOTU	Genus	C/N50 vsCN7 (%)	ANOVA (Welch´s test)
Day 4	Bacteria			
	zOTU2	Burkholderia	-2.31	0
	zOTU3	Massilia	-2.75	0.045
	zOTU76	Methylocella	0.18	0.023
	zOTU191	Opititus	0.06	0.015
	zOTU303	Mesorhizobium	0.05	0.03
	Fungi			
	OTU3	Aureobasidium	-53.7	0.017
	OTU36	Aureobasidium	-1.08	0.02
	OTU1172	Aureobasidium	-0.72	0.034
	OTU/zOTU	Genus	C/N50 vsCN25 (%)	ANOVA (Welch´s test)
	Bacteria			
	zOTU2	Burkholderia	-1.18	0.011
	zOTU18	Undibacterium	-0.91	0.022
	zOTU22	Rhizomicrobium	0.13	0.015

	zOTU64	Leptothrix	0.09	0.049
	zOTU76	Methylocella	0.16	0.041
Time	OTU/zOTU	Genus	C/N50 vsCN7 (%)	ANOVA (Welch´s test)
	Bacteria			
	zOTU57	Candidatus Solibacter	0.49	0.02
	zOTU141	Methylobacterium	0.36	0
	zOTU104	Rhodopila	0.23	0.03
	zOTU1050	Acidocella	0.1	0.04
	Fungi			
	OTU650	Rhodotorula	25.1	0.03
	OTU/zOTU	Genus	C/N50 vsCN25 (%)	ANOVA (Welch´s test)
Day 11	Bacteria			
	zOTU63	Legionella	1.53	0.01
	zOTU141	Methylobacterium	0.35	0.01
	zOTU12	Uncultured_bacterium (Acidimicrobiales)*	0.34	0.01
	zOTU185	Byssovorax	0.28	0.01
	zOTU131	Acinetobacter	0.18	0.02

Table S4: Statistical analysis of functional potential of prokaryotic community (difference between control and treatment; t-test)

	**4 days**					
	**2 % of total C**			**5% of total C**		
	C/N7	CN/25	CN/50	C/N7	C/N25	C/N50
chemoheterotrophy (wo fermentation)	0,590	0,317	0,712	0,041	0,038	0,043
fermentation	0,899	0,181	0,588	0,048	0,029	0,044
chemolitotrophy	0,675	0,023	0,050	0,015	0,017	0,013
photothrophy	0,686	0,381	0,573	0,087	0,123	0,108
methanogenesis	0,625	0,434	0,486	0,356	0,363	0,213
anaerobic metabolism (wo methanogenesis and fermentation)	0,427	0,761	0,704	0,085	0,082	0,101
	11 days					
	2 % of total C			5% of total C		
	C/N7	CN/25	CN/50	C/N7	C/N25	C/N50
chemoheterotrophy (wo fermentation)	0,002	0,151	0,005	0,103	0,054	0,040
fermentation	0,068	0,047	0,008	0,203	0,594	0,181
chemolitotrophy	0,450	0,030	0,042	0,159	0,086	0,384
photothrophy	0,298	0,860	0,803	0,436	0,783	0,589
methanogenesis	0,391	0,391	0,391	-	0,191	0,231
anaerobic metabolism (wo methanogenesis and fermentation)	0,231	0,015	0,026	0,091	0,157	0,081
	25 days					

	2 % of total C			5% of total C		
	C/N7	CN/25	CN/50	C/N7	C/N25	C/N50
chemoheterotrophy<br>(wo fermentation)	0,392	0,305	0,381	0,060	0,003	0,024
fermentation	0,583	0,265	0,690	0,068	0,010	0,372
chemolitotrophy	0,369	0,447	0,319	0,870	0,966	0,958
photothrophy	0,883	0,353	0,248	0,144	0,973	0,848
methanogenesis	0,941	0,894	0,391	0,881	0,960	0,256
anaerobic metabolism<br>(wo methanogenesis and fermentation)	0,411	0,390	0,287	0,087	0,001	0,023

Figure S1 Nitrate N (NO_3) concentration in control samples and samples amended by artificial exudates with different C/N ratios **A/** in concentration of 2 % of total DOC and **B/** in concentration of 5 % of total DOC during 25 days incubation (means, ± standard deviations, n=4)

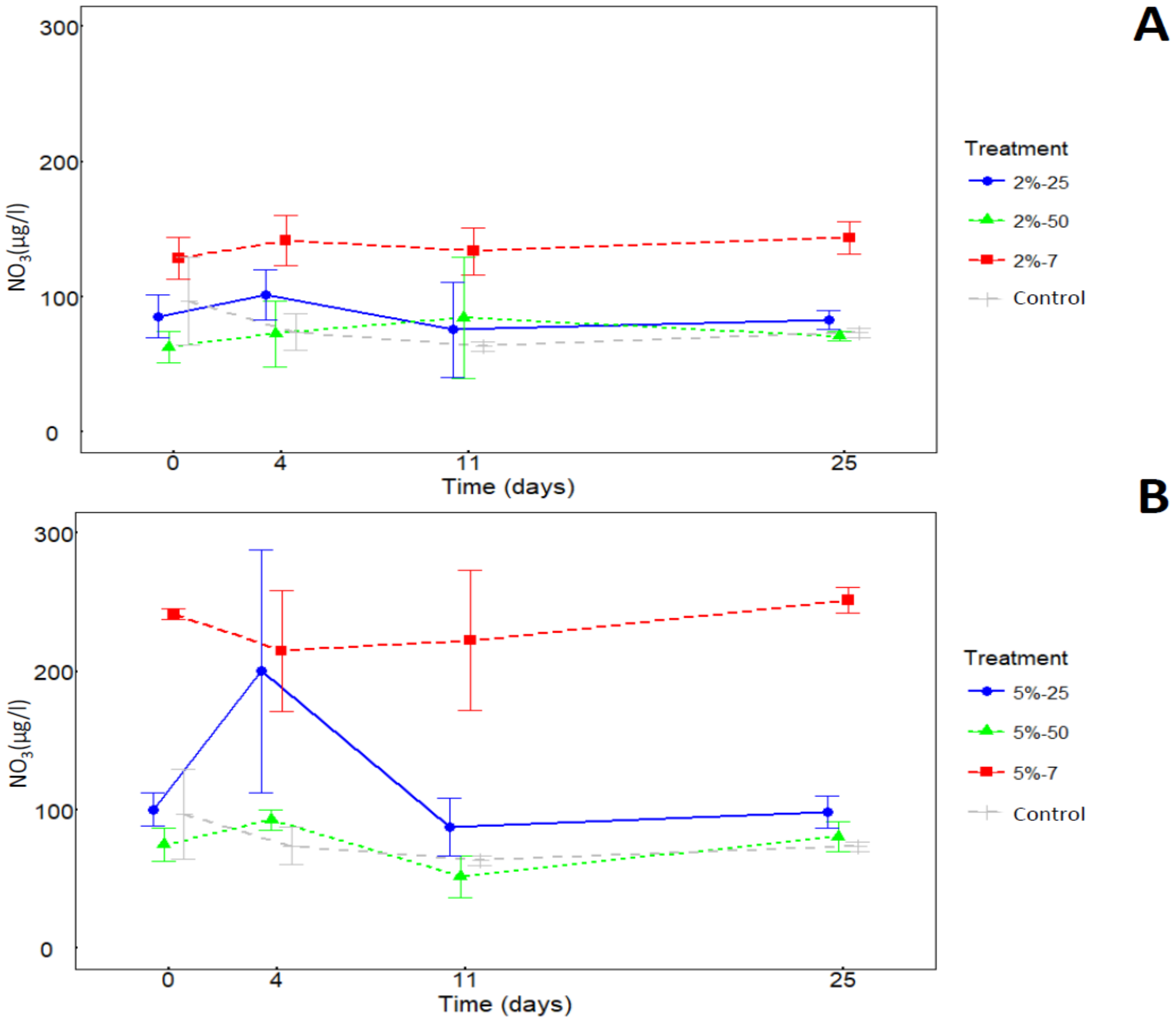
A
B
300
200
100
0
NO_3(µg/l)
0
4
11
25
Time (days)
Treatment
2%-25
2%-50
2%-7
Control
5%-25
5%-50
5%-7
Control

Figure S2 Fungi/bacteria ratio in control samples and samples amended by artificial exudates with different C/N ratios **A/** in concentration of 2 % of total DOC and **B/** in concentration of 5 % of total DOC during 25 days incubation (means, ± standard deviations, n=4)

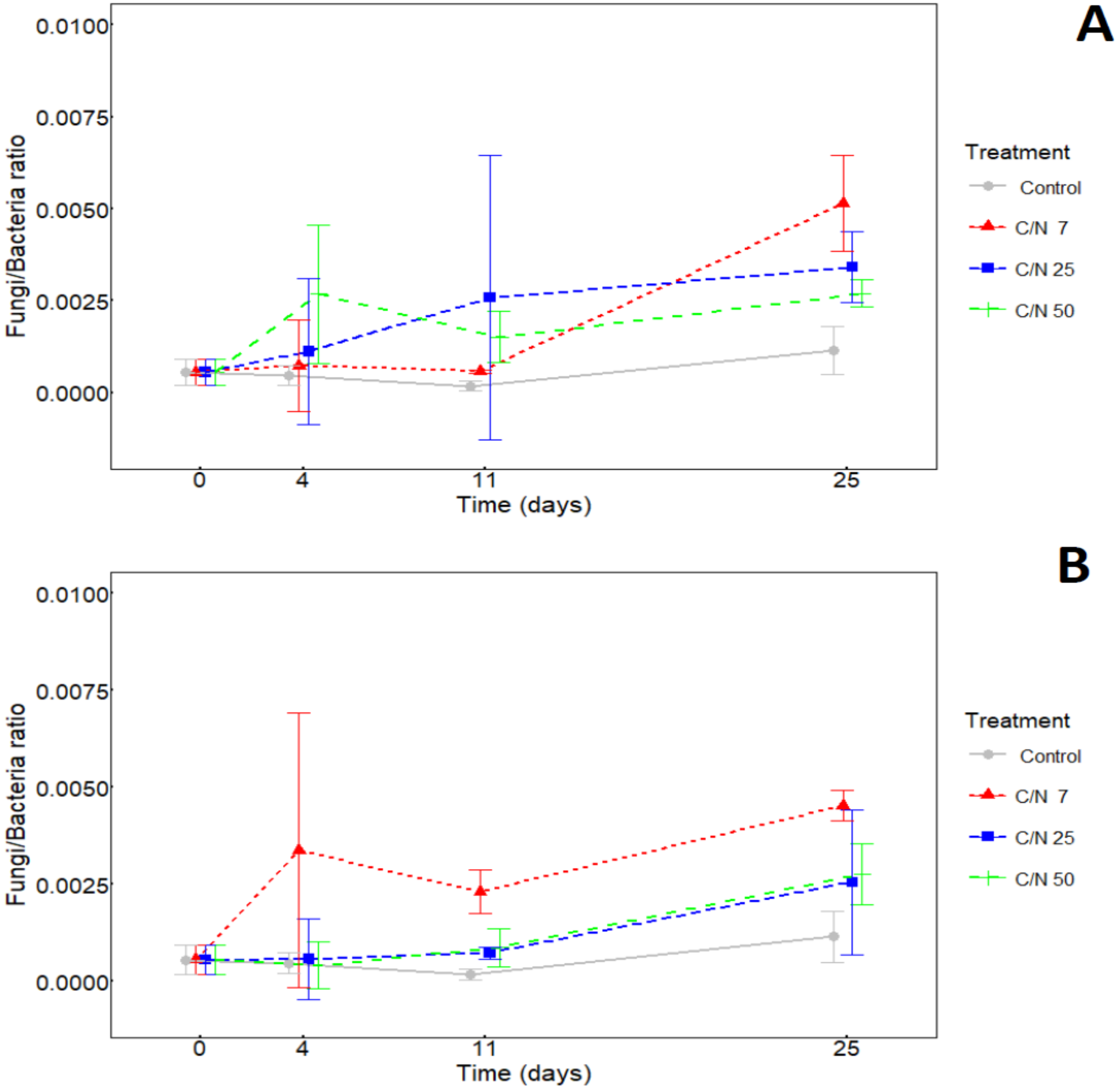

Figure S3 The temporal changes of bacterial community composition **A/** in control and **B/** in samples amended by artificial exudates with different C/N ratios in concentration of 2 % of total DOC and **C/** in concentration of 5 % of total DOC during 25 days incubation

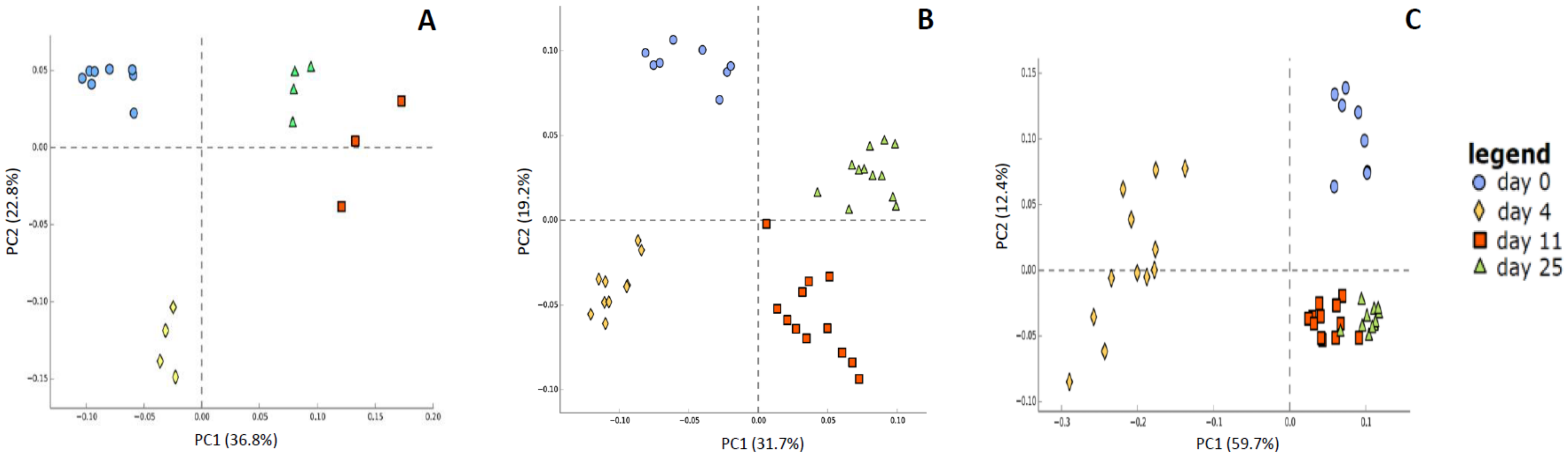

Supplement: Supplementary file 1 — Supplementary Information. [file 41598_2021_97698_MOESM1_ESM.pdf]
